# The mouse genome displays highly dynamic populations of KRAB-zinc finger protein genes and related genetic units

**DOI:** 10.1371/journal.pone.0173746

**Published:** 2017-03-23

**Authors:** Annamaria Kauzlaric, Gabriela Ecco, Marco Cassano, Julien Duc, Michael Imbeault, Didier Trono

**Affiliations:** School of Life Sciences, Ecole Polytechnique Fédérale de Lausanne (EPFL), Lausanne, Switzerland; Imperial College London, UNITED KINGDOM

## Abstract

KRAB-containing poly-zinc finger proteins (KZFPs) constitute the largest family of transcription factors encoded by mammalian genomes, and growing evidence indicates that they fulfill functions critical to both embryonic development and maintenance of adult homeostasis. *KZFP* genes underwent broad and independent waves of expansion in many higher vertebrates lineages, yet comprehensive studies of members harbored by a given species are scarce. Here we present a thorough analysis of *KZFP* genes and related units in the murine genome. We first identified about twice as many elements than previously annotated as either *KZFP* genes or pseudogenes, notably by assigning to this family an entity formerly considered as a large group of *Satellite* repeats. We then could delineate an organization in clusters distributed throughout the genome, with signs of recombination, translocation, duplication and seeding of new sites by retrotransposition of *KZFP* genes and related genetic units (*KZFP/rGU*s). Moreover, we harvested evidence indicating that closely related paralogs had evolved through both drifting and shifting of sequences encoding for zinc finger arrays. Finally, we could demonstrate that the KAP1-SETDB1 repressor complex tames the expression of *KZFP/rGU*s within clusters, yet that the primary targets of this regulation are not the *KZFP/rGU*s themselves but enhancers contained in neighboring endogenous retroelements and that, underneath, *KZFP*s conserve highly individualized patterns of expression.

## Introduction

Dynamic changes in gene super-families are potent drivers of evolution and diversity across species [1, 2]. KRAB-containing zinc finger proteins (KZFPs) constitute the single largest group of transcription factors (TFs) encoded by higher vertebrates, and emerged in a close ancestor of tetrapods some 420 million years ago (mya) [3, 4]. *KZFP* genes were subsequently amplified as the apparent result of adaptive expansion and contraction events [5–7], while being subjected to intense positive selection, so as to constitute today a large repertoire of species-specific TFs [8–12]. This pattern of evolution likely reflects the involvement of KZFPs in the early embryonic repression of endogenous retroelements (EREs), many of which are themselves lineage- or species-restricted [13–15]. Consistent with this hypothesis, the total number of KZFPs encoded by various species somewhat correlates their burden in endogenous retroviruses (ERVs), a class of EREs, and recent waves of ERV invasions have coincided with episodes of *KZFP* genes amplification [16, 17]. As well, the genomes of both humans and mice encode for several hundred KZFPs, yet the pace of expansion of these genes has been higher in the rodent lineage, correlating the persistence in mice, but not in humans, of transposition-competent ERVs. Finally, an arms race model, whereby the host produces a dynamic pool of KZFPs in order to control active EREs that in turn mutate to escape restriction, is supported by evidence retracing the evolutionary history of two human KZFPs and their cognate ERE targets [15].

Canonical KZFPs harbor a highly conserved N-terminal KRAB (Krüppel-associated box) domain [18, 19], responsible for recruiting KAP1/TRIM28 (KRAB-associated protein 1, tripartite motif protein 28) and its associated epigenetic effectors [20–22], and a C-terminal array of zinc fingers (ZNFs), which confers sequence-specific DNA binding potential and is the region where positive selection is observed [5, 23–26]. It seems likely that, after a *KZFP* gene is duplicated through unequal crossing over, gene conversion, or yet other mechanisms, changes in its zinc-finger coding portion can become fixed if they provide the product of this duplication with novel target specificity that benefits the host [27–29].

Here, to investigate the evolutionary path of *KZFP* genes in a species where this family is still subjected to dynamic selective pressures, we explored the mouse genome. This led us to uncover an abundance of yet unreported *KZFP*-related genetic entities in this species [30, 31], to obtain evidence supporting a variety of mechanisms for their expansion, to examine the sequence diversification of closely related paralogs and to unveil a mode of regulation where EREs seem to occupy a prominent place in the transcriptional control of their repressors.

## Results

### The *MMSAT4 satellite* repeat corresponds to *KZFP* genes and *KZFP*-like entities

**Fig 1:**
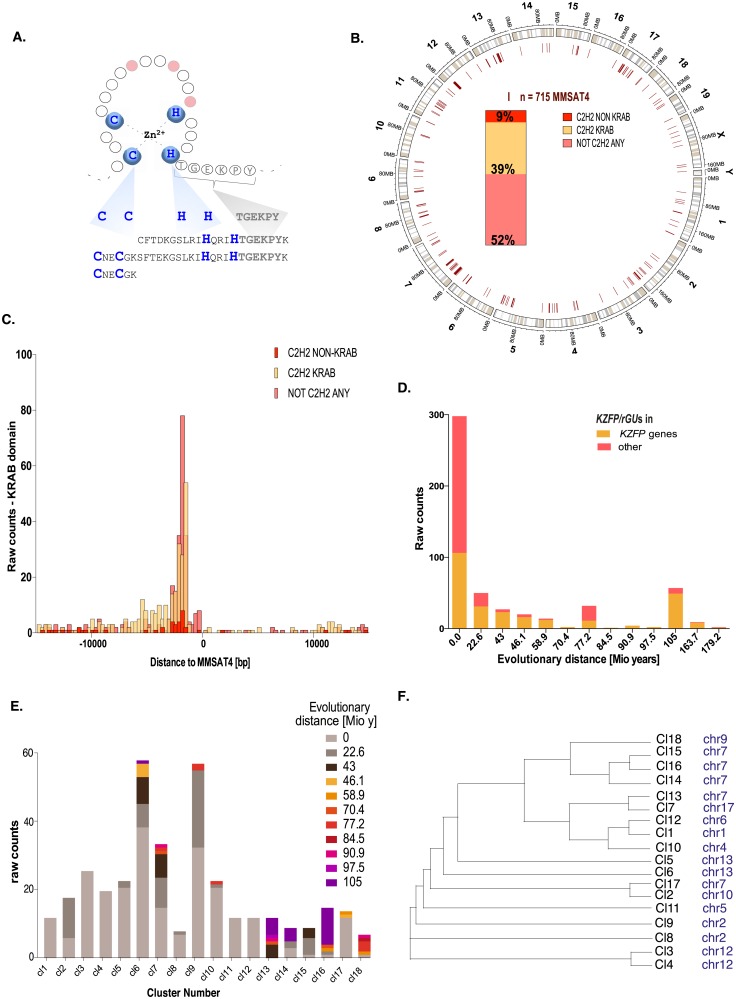
Genomic features and evolutionary origins of murine *KZFP/rGU*s clusters. **(A)** (Top) Schematic representation of a canonical C2H2 zinc finger (ZF) structure, coupled to its 7-amino acids linker, 6 of which (TGEKPY) are highly conserved. The 21–24 amino acid-long ZNF (**C X**_2-4_ **C X**_12_ **H X**_3-4_ **H**) is depicted, with zinc-coordinating cysteines and histidines in blue and putative DNA-contacting residues located at positions -1, 3 and 6 of the alpha helix in red. (Bottom) Consensus sequence of MMSAT4 Satellite Repeat as reported in Repbase, spanning three consecutive but incomplete C2H2 ZNF motifs, each reported on a single line. Highlighted are the canonical cysteine and histidine residues (in blue) and the conserved part of the linker (in grey). **(B)** Circular genomic map displaying the distribution of MMSAT4 elements. Inside the circular plot, stacked bar plot of MMSAT4 elements belonging to C2H2 ZNF protein genes not encoding a KRAB domain, to KRAB C2H2 ZNF protein genes, or to genomic locations not annotated as any type of C2H2 encoding protein genes. **(C)** Positional correlation between MMSAT4 consensus sequences and KRAB-encoding sequences, distinguishing MMSAT4 elements belonging to C2H2 ZNF protein genes not encoding a KRAB domain, to KRAB C2H2 ZNF protein genes, or to genomic locations not annotated as any type of C2H2 encoding protein genes. Genes’ coordinates were extended by 1kb at the 3’ end, and the correlation was calculated over a symmetrical window of 15 kb. **(D)** Raw counts of 518 murine *KZFP/rGU*s shared between phylogenetic branches, belonging to annotated *KZFP* genes or other genetic entities (“other”), with the x-axis representing the evolutionary distances separating the branches. 517/518 of these *KZFP/rGU*s bear a KRAB-encoding sequence within 30 kb upstream of the element itself. **(E)** Age-distribution of *KZFP/rGU*s within clusters. The estimated time of emergence during evolution is color-coded as indicated by the legend. **(F)** Phylogenetic tree of consensus sequences derived per *KZFP/rGU*s cluster, with indication of the chromosome where the relative cluster is located.

The repository of murine repetitive elements lists as a member of the *Satellite* family a simple repeat named ‘MMSAT4’, the consensus sequence of which was derived from arrays of triplets of C2H2 ZNFs located on chromosome 4 (Fig 1A, http://www.girinst.org/repbase/ [32]). However, unlike other *Satellite* repeats, MMSAT4s are not restricted to specific chromosomal positions, as we found 715 of these units spread all over the genome, often concentrated in regions also containing high densities of *bona fide* C2H2 ZNF-coding sequences (Fig 1B). Of the annotated C2H2-protein coding units containing an MMSAT4, the large majority (82%) are canonical *KZFP* genes while a small fraction spans other types of poly-zinc finger protein genes. Conversely, of the currently annotated *KZFP* genes, 87% overlap with MMSAT4 sequences. Additional MMSAT4 elements, making up slightly more than half of the group, fall into previously unannotated sequences (Fig 1B), yet we found them to harbor an upstream KRAB-coding sequence and to coincide with the 3’ end of the cognate transcripts, comparably to canonical KZFP-overlapping MMSAT4s (Fig 1C and S1A Fig). Therefore, MMSAT4s correspond to almost the totality of *KZFP* genes and to additional *KZFP*-like entities. For convenience, we coined the sum of these elements *KZFP/rGU*s, for *KZFP*-related genetic units.

### Evolutionary relationships between *KZFP/rGU*s clusters

Through sequence and synteny analysis of murine *KZFP/rGU*s and their flanking sequences [4], we could assign a putative age to about three quarters of these elements (518/715). We found the vast majority to be mouse-restricted, whether *KZFP* genes or pseudogenes and non-annotated entities, and very few to have human orthologs, corresponding to an evolutionary distance of 90.9 million years ago (mya) (Fig 1D). By setting a cutoff of at least 8 MMSAT4 elements less than 500 kilobases (kb) apart, we could further define 18 clusters distributed amongst 11 chromosomes (S1 Table). Some clusters contained both mouse-specific and evolutionarily older *KZFP/rGU*s (e.g. clusters 14 and 16 on chromosome 7), yet none was entirely constituted of highly conserved members, and 5 of the 18 predefined clusters contained exclusively mouse-restricted *KZFP/rGU*s (Fig 1E).

Consistent with previously proposed mechanisms of *KZFP* genes amplification [7], we observed that murine *KZFP/rGU*s shared higher sequence homology within a cluster than amongst spatially unrelated elements (S1B Fig). We thus derived a consensus sequence for each cluster and built a tree based on their degree of similarity. This confirmed that clusters located on a same chromosome were usually more closely related. However, there were exceptions to this trend. For instance, clusters 1 and 12, respectively on chromosomes 1 and 6, were highly homologous, as were clusters 2 and 17, on chromosomes 10 and 7. This strongly suggests that large segment duplications, followed by chromosomal rearrangements, have contributed to the expansion of *KZFP/rGUs*, as previously postulated for canonical *KZFP* genes [5]. Interestingly, clusters partially composed of conserved *KZFP/rGU*s, such as number 14, 16 and 18, segregated away from the others (Fig 1F). Careful examination revealed age-independent clustering of *KZFP/rGU*s within these clusters (S1C Fig).

When further studying *KZFP/rGU*s clusters, we very rarely found genes other than *KZFP*s. The murine genome harbors several million loci derived from transposable elements (TEs), and when examining the density of these units within clusters of *KZFP/rGU*s we found it to be no different than elsewhere in the genome (S1D Fig). However, in *KZFP/rGU*s clusters, we found a marked enrichment in members of two subgroups of ERVs, ERVK and ERV1 (S1E Fig). Similar to *KZFP*s, olfactory and vomeronasal receptor genes (*OLFR* and *VMNR* genes, respectively) have been amplified through rounds of segmental duplications while undergoing positive selection [33, 34]. However, we found neither 14 clusters of *OLFR* genes nor a cluster of *VMNR* genes, each defined as a group of at least 10 such genes situated less than 500 kb apart, to display similarly biased TE distribution (S2 Table, S1F Fig). *KZFP/rGU*s clusters thus exhibit a particular TE content when compared to the rest of the genome or to other gene super-families amplified by related mechanisms.

**Fig 2:**
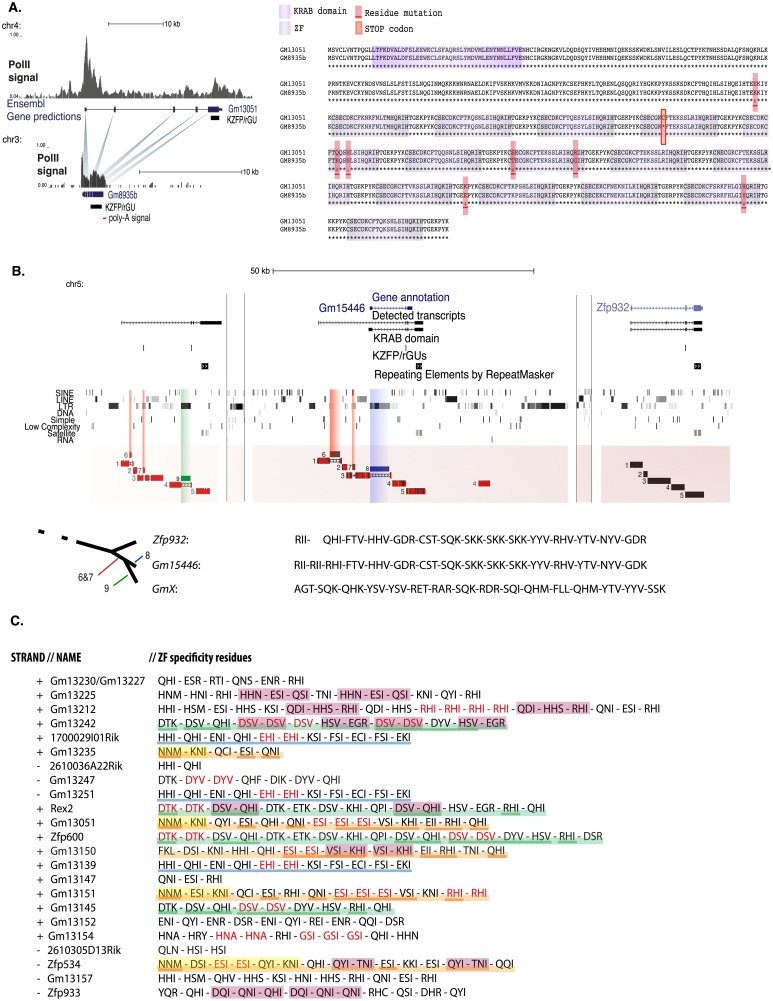
Evolutionary relationships within the *KZFP-rGU*s superfamily. **(A)** (Left) Schematic representation of how retrotransposition of the *KZFP/rGU Gm13051* (located on chromosome 4) may have yielded the *Gm8935b* retrogene (on chromosome 3, to which we gave a different annotation than the one deposited in Ensembl as *Gm8935*, based on similarities with its putative donor gene). ChIP-seq density signal of RNA polymerase II (PolII) at the corresponding loci and genomic structures of *Gm13051* and *Gm8935b* are shown, with thick lines depicting exons and thin lines intronic or non-transcribed sequences. *KZFP/rGU*s (black) and poly-A sequences (red) are annotated separately. (Right) Amino acid sequence comparison of putative GM13051 and GM8935b proteins. KRAB and ZNF domains are shaded in dark and light purple, respectively. Orange rectangle, location of stop codon in GM8935b; red rectangle, mutations located in 3’ UTR. (*) indicates full conservation, (:) amino acids with highly similar properties and (.) unrelated residues. **(B)** (Top) UCSC Genome Browser view of a genomic region containing *KZFP/rGU*-related genes *GmX* (annotated in present study), *Gm15446*, and *Zfp932*, located in cluster 11 on chromosome 5. In order from the top, tracks for: genes annotated in RefSeq, our de-novo transcripts annotation, KRAB-encoding sequences, *KZFP/rGU*s, and repeats as reported in RepeatMasker. BLAT results for segments 1–5 of *Zfp932* over the studied region are shown, with red lines indicating single differences in homologous sequences. (Center) Segments 6 and 7 of *Gm15446*, together with their BLAT results over *GmX*, are highlighted by red vertical bars. Segments 8 and 9, belonging to *Gm15446* and *GmX* are highlighted by blue and green vertical bars, respectively. Homology interruptions of segments 1, 3 and 4 are depicted by hollow bars. Lengths of DNA stretches between genes are not to scale. (Bottom) Putative evolutionary tree of *Zfp932*, *Gm15446* and *GmX*, with their zinc finger signature, that is, for each ZNF the three major DNA-contacting residues. **(C)** Strand, name of the corresponding annotated gene and ZNF specificity residues of *KZFP/rGU*s for which a related transcript was detected in cluster 10. Elements are reported in linear order of appearance in the cluster. Each ZNF, represented by the triplet of its DNA-contacting amino acids, is separated from the others by a dash. Red characters indicate tandem duplications of a given triplet of amino acids. Colored underlining highlights triplets conserved in multiple putative and bona-fide *KZFP* genes, to mark potential paralogs and closely related genes or pseudogenes. Pink shading marks triplets repeated multiple times, in the same order, within one *KZFP* gene or pseudogene. Yellow shading marks the first triplets of highly homologous genes *Gm13235*, *Gm13051*, *Gm13151*, *Zfp534*, exemplifying the variability of divergence patterns observed within a cluster.

When examining *KZFP/rGU*s not situated in clusters, we identified several instances where they most likely resulted from retrotransposition, as indicated by the absence of introns, the presence of a poly-A-coding sequence at their 3’ end and their homology with intron-containing cluster-located elements (Fig 2A, left panel). Although they were situated on other chromosomes than their source element, such retrotransposed *KZFP*s displayed signs of positive selection. This was exemplified by the newly annotated pseudogene *Gm8935b*, where a sharp increase in mutations density was found 3’ of a premature STOP codon gained in the processed pseudogene, compared to its apparent donor gene. Moreover, we detected a distinctive enrichment in RNA-polymerase II over *Gm8935b* (Fig 2A, right panel). These observations led us to conclude that *KZFP/rGU*s resulting from retrotransposition events can be transcriptionally active and that, when positively selected, these elements could contribute to seeding new *KZFP/rGU*s clusters.

### Sequence evolution of *KZFP/rGU* paralogs

The analysis of *KZFP/rGU*s revealed that individual units accumulated various degrees of mutations, some of which compromised their coding potential. However, of 1306 C2H2-encoding transcripts that could be assigned to *KZFP/rGU*s in murine embryonic stem cells (mESC), 734 mapped to entries of the Ensembl listings of genes, accounting for multiple isoforms of a totality of 252 genes. Of those, more than three quarters (196/252) corresponded to *bona fide KZFP* genes, 13% (34/252) to non-KRAB *ZFP* genes and 9% (22/252) to other genes. Of 572 novel transcripts, 490 coded for a stretch of C2H2 zinc fingers preceded by a KRAB domain in the same reading frame and without intervening STOP codon (S1 File, S3 Table). This suggests that positive selection is acting to preserve the functional core of most of these elements.

Retracing the full evolutionary history of *KZFP/rGU* clusters remains a complicated task, due to both persistent retrotransposition activity of many mouse TEs and confounding effects of partially overlapping genomic rearrangements (S2B Fig). We thus designed an additional approach to date the emergence of mouse-specific and closely related *KZFP/rGU*s. For this, we focused on the regions directly bordering *KZFP/rGU*s and studied their sequence conservation after splitting them in segments, which we considered as separate units. Coupling our analysis to newly annotated *KZFP/rGU*s-containing transcripts helped us circumscribe the region of interest to the one enclosed by gene borders, although segments numbers and limits were arbitrarily fixed. As an example, cluster 11 contains three *KZFP/rGU*s: *Zfp932*, *Gm15446* and a putative gene not annotated in the RefSeq nor in other genes repositories, which we named *GmX*. *Zfp932* and *Gm15466* are known paralogs, recently characterized through genomic and functional studies as responsible for recognizing partly overlapping sets of TEs [35]. By defining discrete segments of DNA within the regions spanning *Zfp932* and *Gm15466*, we could determine by BLAT [36] that the next most conserved sequence was spanning *GmX*. Taking *Zfp932* (Fig 2B, segments 1–5) as main viewpoint allowed us to single out a series of new segments (Fig 2B, segments 6–9) that disrupted homology between the three genes. Segments 6–7 were present in *Gm15446* and *GmX*, but not in *Zfp932*, while segment 8 was restricted to *Gm15446* and segment 9 to *GmX*. Furthermore, several nucleotide differences were found within the studied segments of paralogs *Zfp932* and *Gm15446*, and even more when comparing them to *GmX*. Taking into account the sum of these observations, we could draw a tree representing the likely sequential emergence of these genes, with *Zfp932* followed by *Gm15446* and then *GmX*. Interestingly, the ZNF signature of *GmX* strongly differs from those of *Zfp932* and *Gm15446*, which are highly similar. Accordingly, segment 5, which spans the *KZFP/rGU* of each of these three genes, was the least conserved within the whole cluster. A broader examination of the specificity residues, that is, of the amino acids at positions -1, 3 and 6 of the DNA-contacting alpha helix, encoded by *KZFP/rGU*s of cluster 10 similarly revealed complex mechanisms of diversification of neighboring poly-zinc fingers, with evidence for duplications, deletions, inversions and mutations of ZNF-coding sequences. Triplets of the DNA-contacting residues belonging to one ZNF could be duplicated in tandem up to 4 times, and combinations of 2 to 7 sequential triplets could be repeated within the same gene or pseudogene. These repetitions were different between highly homologous signatures, which were not residing in adjacent *KZFP/rGU*s and could even be encoded on the opposite strand (Fig 2C). Together, these data support the model of a high level of plasticity within poly-zinc fingers coding sequences, with fast emergence of new zinc fingerprints, which either get fixed by positive selection or lose their coding potential and degenerate.

### Transcriptional regulation of *KZFP/rGU*s

**Fig 3:**
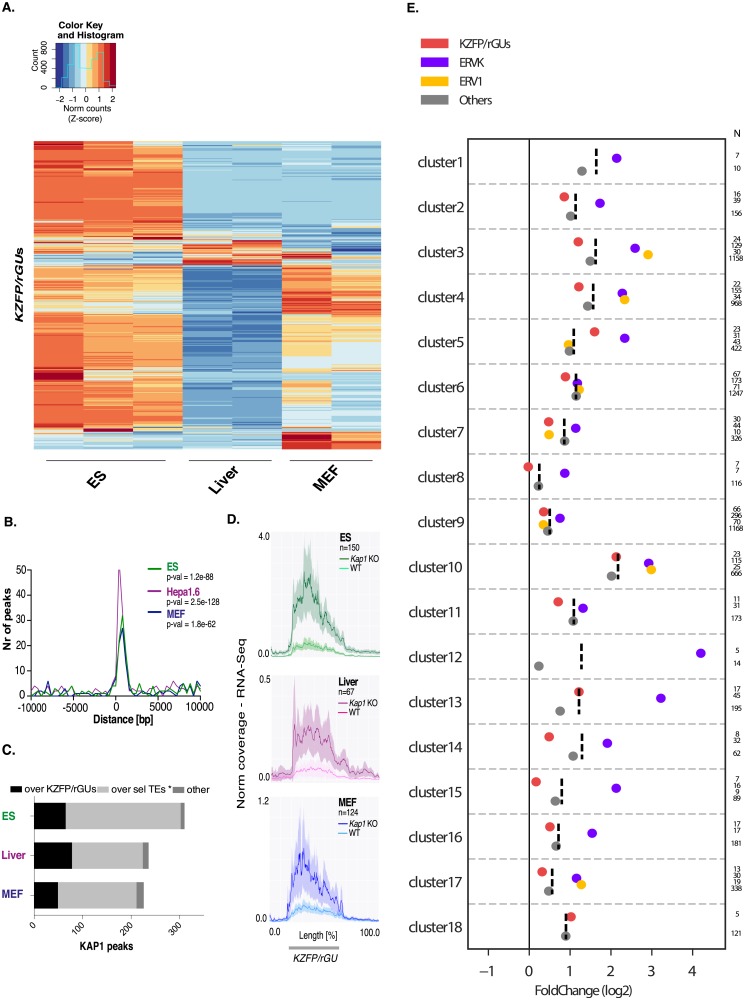
KAP1 binds to *KZFP/rGU*s and neighboring TEs. **(A)** Heatmap of RNA-Seq depicting expression levels of *KZFP-rGU*s-derived transcripts in WT mES cells, liver cells and MEFs. Exact values were reported in S4 Table. **(B)** Positional correlation between *KZFP/rGU*s and KAP1 peaks in mES cells, Hepa 1.6 cells and MEFs, over a window of 10 kb. In the legend, indication of the p-values obtained by Fisher’s exact test. **(C)** Distribution analysis of KAP1 peaks located within *KZFP/rGU*s clusters per tissue. Selected TEs (“Sel. TEs”) comprise repeats annotated in RepeatMasker excluding Satellite, Simple, Low Complexity, Unknown repeats and subgroups counting less than a 100 hits among all *KZFP/rGU*s clusters. Peaks overlapping yet other entities, for instance genes, are listed as “other”. **(D)** Normalized RNA-Seq average coverage over significantly dysregulated *KZFP-rGU*s, considering a flanking region of 1.5 kb upstream and 3.5 kb downstream of each element, in mES (top), liver (center) and MEF (bottom) WT and *Kap1* KO cells. Shaded curves represent the 95% confidence interval around the mean. **(E)** Mean log2 of the expression fold change upon *Kap1* removal in mES cells of elements present in *KZFP/rGU*s clusters: *KZFP/rGU*s, ERVKs, ERV1s and the rest of TEs (“others”) (dots), as well as their total average (dashed line). The analysis was performed per cluster, with the number of elements in each category reported on the right (categories with fewer than 5 elements were not considered).

We next assessed by RNA-Seq the transcription of *KZFP/rGU*s in three distinct murine cell types: mESC, hepatocytes and MEFs. Each displayed a specific pattern of *KZFP/rGU* expression, with mESC transcribing the highest number of elements (Fig 3A, S4 Table). The ZNF-coding region of *KZFP* genes was previously demonstrated to recruit KAP1, and to be secondarily subjected to trimethylation of histone 3 on lysine 9 (H3K9me3) [37, 38]. ChIP-seq analyses confirmed the extensive binding of KAP1 at *KZFP/rGU*s in mESC, MEF and Hepa-1.6, a murine hepatoma cell line that we used as a surrogate for hepatocytes (Fig 3B). The density of KAP1 peaks correlated with that of *KZFP/rGU*s over many clusters in all three tissues, including clusters completely devoid of annotated protein-coding genes (S3A–S3C Fig). However, *KZFP/rGU*s themselves only accounted for a fifth to at most a third of all of KAP1 peaks within these regions, where they were largely outnumbered by TEs (Fig 3C).

Upon *Kap1* knockout (KO), many *KZFP/rGU*s were upregulated in all three tissues, albeit with some differences: in mESC, 145 out of 150 dysregulated elements increased in expression, while for liver and MEF the ratios were 66/67 and 77/124, respectively (Fig 3D). Noteworthy, *KZFP/rGU*s upregulation upon KAP1 depletion was significant in mESC and liver only for elements situated within clusters, but not for their more isolated counterparts, while in MEF it was significant in neither setting (S3D Fig). We explored more in depth the apparently collective behavior of *KZFP/rGU*s located within a same cluster upon KAP1-depletion. For this, we scrutinized cluster 10, which contained the highest fraction of upregulated elements in these systems (23/25 in mESC, 15/25 in liver and MEF). We first found that, in spite of a high degree of homology between these *KZFP/rGU*s, their promoter regions were markedly divergent (S3E Fig). Second, only a third of these units (8/25) were deregulated in all three tissues, all the others behaving in a cell-specific manner. *KZFP/rGU*s transcription, as its perturbation, was not restricted to elements that were part of previously annotated protein-coding genes, and it always occurred in the sense orientation, indicating that transcriptional read-through from other units, which would have contributed both sense and antisense transcripts, was not significantly affecting our analysis (S3F Fig).

Noteworthy, along with the higher density of KAP1 peaks measured within *KZFP/rGU*s clusters compared to *OLFR* and *VMNR* genes clusters, we found an important frequency of upregulated TEs in *Kap1* KO tissues (S3G Fig). TEs are subjected to KAP1-mediated repression via sequence-specific tethering by KZFPs [15, 35, 39–41]. Elements of the ERVK and ERV1 subgroups were found to be preferential sites of KAP1 recruitment within *KZFP/rGU*s clusters, but other TEs and *KZFP/rGU*s were equally upregulated upon KAP1 removal (Fig 3E and S3H and S3I Fig), indicating that de-repression then extended to the whole cluster when KAP1-targeted TEs lost the regulator.

### Mechanisms of *KZFP/rGU*s clusters control

**Fig 4:**
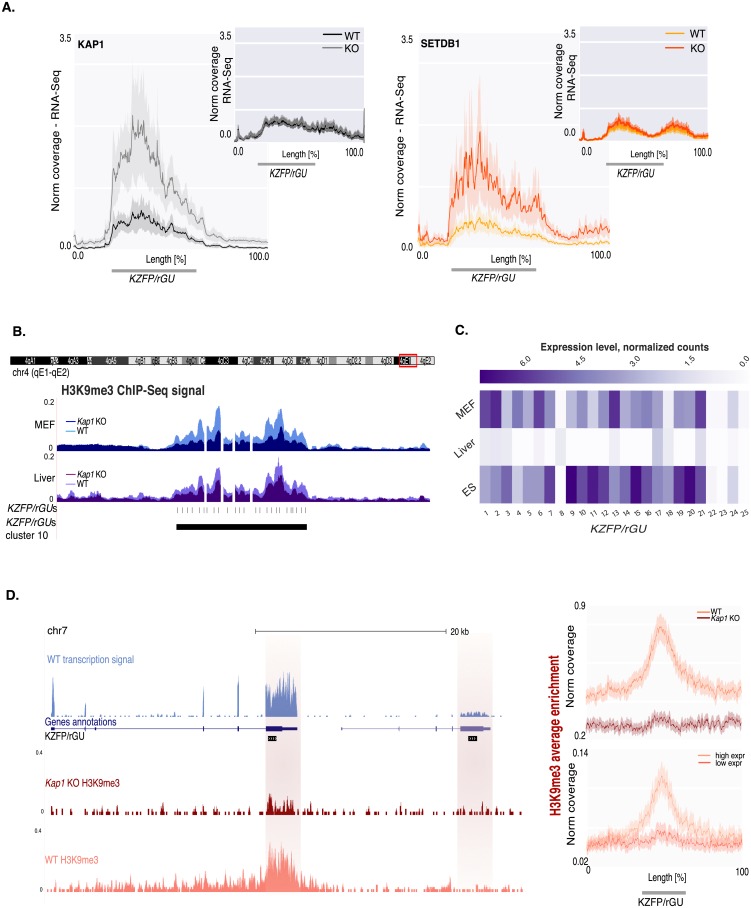
KAP1 and SETDB1-mediated transcriptional regulation of *KZFP/rGU* clusters. **(A)** Normalized RNA-Seq average coverage over *KZFP-rGU*s, considering a flanking region of 1.5 kb upstream and 3.5 kb downstream of each element. Shaded curves represent the 95% confidence interval around the mean. RNA-Seq data is plotted for *KZFP/rGU*s upregulated (main panels) or unaffected (smaller top right inserts) in either *Kap1* (left) or *Setdb1* (right) KO ES cells. **(B)** UCSC Genome Browser view of H3K9me3 ChIP-Seq profiles over *KZFP/rGU*s in cluster 10 in WT and *Kap1* KO cells (light and dark color shades, respectively) for MEFs (top track), and liver cells (bottom track). Underneath, tracks for single *KZFP/rGU*s and for clusters are shown. **(C)** Heatmap illustration of RNA-Seq signal over *KZFP/rGU*s in cluster 10, reported linearly in their genomic order, for MEFs, liver and ES cells. **(D)** (Left) UCSC Genome Browser view of two *KZFP* genes with RNA-Seq profiles of WT MEFs (blue) and H3K9me3 of WT and *Kap1* KO MEFs (red and pink, respectively). Below the RNA-Seq profile, genes annotated in RefSeq and the *KZFP/rGU*s tracks are displayed. The 3’ end of *KZFP* genes is highlighted by red-shaded vertical bars. (Right) Normalized H3K9me3 ChIP-Seq enrichment over (upper panel) *KZFP/rGU*s in WT and *Kap1* KO MEFs, and (lower panel) *KZFP/rGU*s highly and lowly expressed in WT MEFs, including 5.5 kb on each side of *KZFP/rGU*s.

The histone lysine methyltransferase (KMT) SETDB1, a mediator of KAP1-induced repression, was also enriched at *KZFP/rGU*s, where its recruitment markedly dropped upon *Kap1* knockdown, in agreement with previous findings for canonical *KZFP* genes [38] (S4A Fig). Furthermore, the same range of *KZFP/rGU*s was upregulated in *Kap1* and *Setdb1* KO mESC (Fig 4A), indicating that the transcriptional dysregulation recorded at these loci was likely mediated by the canonical KAP1-SETDB1 complex, known to lead to H3K9me3 deposition and silencing of underlying elements [21, 37, 42–44].

Although KAP1-dependent deposition of H3K9me3 could be so extensive as to appear as covering entire *KZFP/rGU*s clusters both in MEF and liver, the underlying elements were actively transcribed (Fig 4B). Remarkably, neighboring elements within a cluster could display from rather homogeneous to very dissimilar expression levels in a given tissue, and the same element could be highly expressed in one cell type and barely detectable or completely silenced in another (Fig 4C). A closer examination revealed that H3K9me3 was not homogeneously distributed over clusters, being very high over the 3’ end of *KZFP* genes and *KZFP/rGU*s but practically absent from their promoters (Fig 4D, left). Loss of H3K9me3 upon *Kap1* removal was observed over the entire cluster, including every 3’ end of *KZFP/rGU*s (Fig 4D, top right). Nevertheless, there was no correlation between its loss and the upregulation of the underlying element in *Kap1* KO cells, and this mark was particularly abundant over highly transcribed *KZFP/rGU*s (Fig 4D, bottom right, S4C Fig).

**Fig 5:**
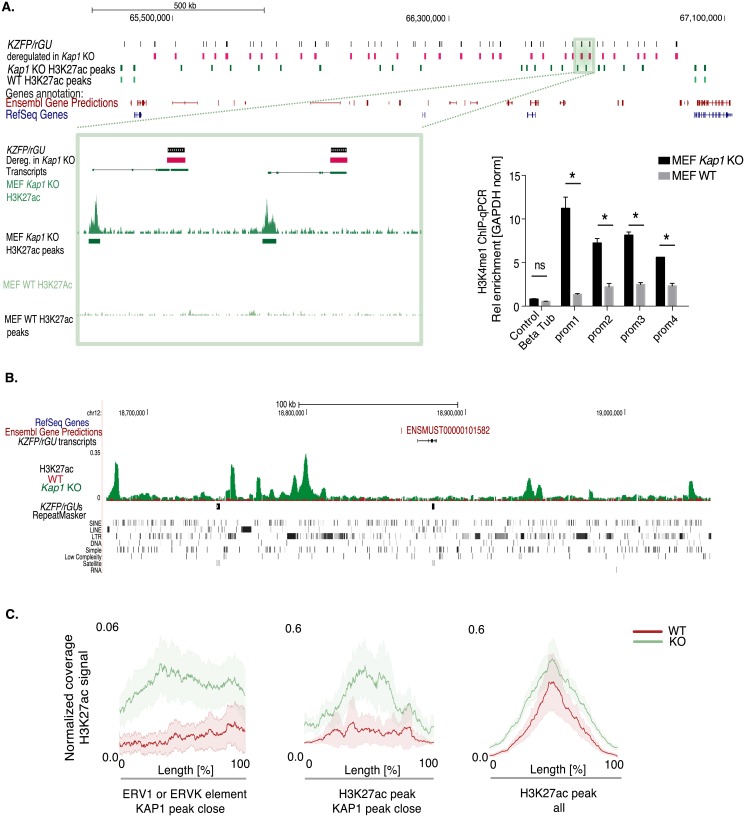
KAP1 influence at *KZFP/rGU* clusters. **(A)** (Top) UCSC Genome Browser view of a region containing a high density of *KZFP/rGU*s (black bars), many of which are dysregulated in MEF *Kap1* KO cells (pink bars). From the top, tracks for *KZFP/rGU*s, H3K27ac peaks in MEF WT and *Kap1* KO cells (light and dark green bars, respectively), and genes annotated in Ensembl and RefSeq are displayed. (Bottom, left) zoom-in of H3K27ac ChIP-Seq profiles and peaks upstream of two distinct *KZFP/rGU*s upregulated upon *Kap1* deletion. Transcripts annotated in the frame of this study are reported below the track of dysregulated *KZFP/rGU*s. (Bottom, right) ChIP-PCR of H3K4me1 over several newly revealed TSSs within this region in MEF WT and *Kap1* KO cells. Enrichments are normalized to those over *Gapdh* promoter, *Beta-Tubulin* being an additional promoter whose transcription is not affected by *Kap1* removal (control). **(B)** UCSC Genome browser views of H3K27ac profiles in MEF (superimposing WT in red and *Kap1* KO in green) over part of *KZFP/rGU*s cluster 3. From top, tracks for genes annotated in Ensembl and RefSeq, de-novo *KZFP/rGU*s transcripts annotation, H3K27ac ChIP-Seq profiles, *KZFP/rGU*s and repeats as reported by RepeatMasker. Genomic stretches gaining H3K27ac signal upon *Kap1* KO do not overlap with any TSS. **(C)** H3K27ac ChIP-Seq normalized average coverage in WT (red) and *Kap1* KO (green) MEFs over: *left*, ERV1 and ERVK elements located within *KZFP/rGU*s clusters and less than 3 kb away from a KAP1 peak in the same tissue; *center*, H3K27ac peaks within *KZFP/rGU*s clusters, not matching any *KZFP/rGU* transcript nor annotated promoter, less than 3 kb away from a KAP1 peak; *right*, without any restriction for KAP1 peak proximity. Shaded curved represent the 95% confidence interval around the mean.

**Fig 6:**
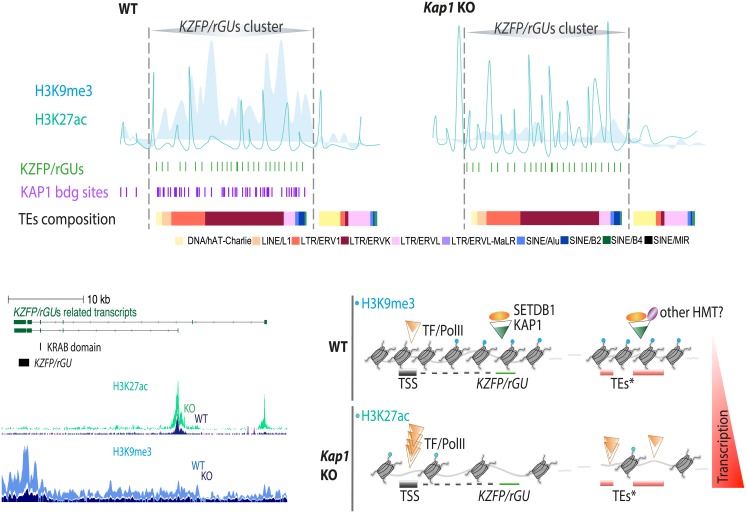
Model for transcriptional regulation of *KZFP/rGU*s clusters. (Top) Schematic representation of *KZFP/rGU*s clusters main features in WT and *Kap1* KO cells. From top: *KZFP/rGU*s cluster limits, profiles of H3K9me3 and H3K27ac enrichment (full and hollow curves, respectively); *KZFP/rGU*s, showing the characteristic local high density of elements; KAP1 peaks, correlating with the *KZFP/rGU*s-dense region; track of TEs. (Bottom, left) an example illustrating the precise distribution in WT and *Kap1* KO cells of H3K27ac and H3K9me3 profiles, and their correspondence with to *KZFP/rGU*-derived transcripts, where thicker lines represent coding sequences and thin lines non-coding sequences, with arrows indicating directionality of the transcripts. KRAB-encoding sequences and *KZFP/rGU*s are outlined separately, below the annotated transcripts. (Bottom, right) Molecular model of KAP1-mediated regulation of *KZFP/rGU*s clusters. In WT cells, the complex KAP1-SETDB1, possibly coupled to other HMTs, binds *KZFP/rGU*s and discrete sets of TEs. *KZFP/rGU*s accumulate H3K9me3 at their 3’end, but their promoter is devoid of this mark and can be bound by transcription factors and the RNA-polymerase II machinery, generating transcripts (higher panel). Upon *Kap1* deletion, SETDB1 is no longer recruited, H3K9me3 levels drop, and H3K27ac becomes enriched over TEs and promoters allowing for a general increase in transcription, albeit still under the differential influence of specific transcription factors (lower panel).

We thus examined the impact of KAP1 depletion on the prevalence of H3K27ac, a histone mark associated with active promoters and enhancers, within *KZFP/rGU*s clusters. In clusters where a high fraction of *KZFP/rGU*s were dysregulated upon *Kap1* KO in MEF cells, many new sites became enriched for this mark, most of which were just upstream of previously unidentified transcriptional start site (TSS, Fig 5A, left). ChIP-qPCR for H3K4me1, another histone modification marking actively transcribed promoters, confirmed the activation of numerous *KZFP/rGU*s genes in these clusters upon *Kap1* deletion (Fig 5A, bottom right panel). Other regions within *KZFP/rGU*s clusters, not corresponding to any TSS, gained H3K27ac (Fig 5B and S5A Fig). These did not overlap with *KZFP/rGU*s themselves, but with TEs from all subclasses (S5B Fig). Upon KAP1 depletion, H3K27Ac was notably, but not exclusively, enriched at ERV1 and ERVK integrants normally situated in the vicinity of a KAP1 binding site (Fig 5C). Removal of KAP1 thus appeared to unleash enhancers, many located within TEs, with secondary transcriptional activation of *KZFP/rGU*s and other TEs situated nearby (Fig 6). Of note, proximity to a KAP1 binding site was not critical for the dysregulation of *KZFP/rGU*s, indicating that the regulatory mechanisms acting on these units are long-range (S6A Fig). Moreover, the transcriptional dysregulation of *KZFP/rGU*s was fully reversible upon re-expressing *Kap1* in *Kap1* KO MEF, but their upregulation was comparable or more pronounced immediately after deletion than at later times, suggesting that after long-term culture some compensatory mechanisms could dampen the transcriptional phenotype of *Kap1* KO cells (S6B and S6C Fig). Upregulation of *KZFP/rGU*s upon *Kap1* removal was confirmed on cDNAs generated by priming with random hexamers, suggesting that Poly(A)- transcripts, known to generate from *ZFP* genes [45], are not following a different transcriptional regulation compared to their Poly(A)+ counterparts (S6C Fig).

## Discussion

The majority of present members of the murine *KZFP/rGU*s family emerged after the mouse-rat split and is organized in clusters of sequence-related elements. The genomes of mice and close ancestors appear to have constituted a particularly favorable ground for the expansion of *KZFP/rGU*s, as their number in this species far exceeds what is found in most other higher vertebrates [4]. Most murine *KZFP/rGU*s clusters include mainly such recent elements. Moreover, more evolutionary conserved clusters also contain young *KZFP/rGU*s, indicating that they too have been subjected to recent expansion. We presume that the sequence relatedness of clusters located on distinct chromosomes results from new genetic rearrangements, some of which may have been initiated by retrotransposition events. The majority of mouse *KZFP/rGU*s being specific of this species, comparative genomics could not be used to explore further the evolutionary relationships between these elements. Examining sequence conservation solely at the level of these units also proved rather uninformative, as were neighboring coding sequences because most *KZFP/rGU*s clusters are devoid of genes other than *KZFP*s. Therefore, we decided to study the local sequence divergence, limiting the region of interest to the transcript borders and dividing it into smaller segments which were examined individually and as a bloc. This approach consolidated the model of an expansion of *KZFP/rGU*s clusters, after initial seeding of a chromosomal locus, via gene and segment duplication [5, 7, 11, 46]. The mechanisms driving these waves of expansion remains to be formally established, although we observed an enrichment of discrete families of EREs, ERV1 and ERVK, within *KZFP/rGU*s clusters. Whether this is causally linked or purely coincidental cannot be determined, yet it is remarkable that the ancestral mouse genome was massively targeted by these ERVs between 80 and 50 mya (for ERV1) and 50 and 30 mya (for ERVK) [47], that is at the same time as *KZFP/rGU*s expanded.

Comparing neighboring *KZFP/rGU*s within a cluster revealed signs of both genetic drift and genetic shift concentrated on their ZNF-coding sequences. On the one hand, paralogs drifted by progressively accumulating non-synonymous mutations in these regions, on the other hand their putative DNA binding specificity could altogether shift by insertion of new ZNF-coding segments. *KZFP*s and EREs appear to be engaged in an evolutionary arms race, and the burden of EREs correlates with the number of *KZFP*s in 16 mammalian genomes [16]. It could be that clusters counting more elements target a particularly conspicuous group of EREs, or possibly closely related groups of EREs, as recently observed [35]. Alternatively, mutations within a cluster could confer to its components specificity to very diverse classes of EREs and other genetic elements, and over-amplification of one cluster compared to others could be partially due to a higher degree of plasticity of its genomic locus. Co-option in the transcriptional control of genes and other cellular functions also likely explain the fixation of a significant fraction of *KZFP*s [4, 15].

Expression of *KZFP* genes, like that of other transcription factors, is selective and generally low, whether in undifferentiated cells or in somatic tissues [11]. It is thought that this allows controlling the number of sites bound genome-wide by a KZFP at any given time, and to limit its recruitment at imperfect target sequences [17]. Here, we observed that, within *KZFP/rGU*s clusters, transcriptional control of *KZFP* genes was partly achieved through KAP1-mediated taming of enhancers contained in neighboring EREs. Of note, KAP1 also binds to the 3’, ZNF-coding end of *KZFP* genes, but from this location we found it to exert no repressive effect on their expression, as previously noted [48]. It suggests that at these locations the master regulator, associated effectors and resulting histone marks play different roles. Preventing recombination within these highly repetitive sequences has been suggested [37, 49], but it is unclear how this alone would explain that KAP1 be particularly abundant on highly expressed *KZFP/rGU*s within a cluster. It could thus be that KAP1 recruitment at the 3’ end of these units contributes to three-dimensional topology of *KZFP* clusters, which display a peculiar chromatin organization with accumulation of both repressive and active histone marks [49–51].

ERV-contained enhancer sequences serve as tissue-specific transcription modulators [52–55] and KAP1 control over such cis-acting elements was documented in undifferentiated and somatic cells [56, 57]. KAP1-mediated repression of ERV enhancers seems to impact on all *KZFP/rGU*s, at least based on the global upregulation of these units when it is knocked out. However, even closely located elements within a cluster exhibit exquisitely individualized regulation, as illustrated by their often highly tissue-specific patterns of expression [58]. On the one hand, this could reflect the recognition of their promoters by equally tissue-specific activators. On the other, it could result from the loss of KAP1 at selected ERV enhancers, following the low expression in a given tissue of particular KAP1-tethering KZFPs responsible for the sequence-specific recognition of these ERVs. Irrespective of the underlying mechanism, this rapid divergence in the transcriptional control of closely related paralogs is yet another evidence for the multifaceted roles accomplished this remarkably plastic family of gene regulators.

## Material and methods

### Cell culture and mouse work

mESCs and MEFs wild-type (WT) and KO for *Kap1* were cultured and generated as previously described [57, 59] (strain C57BL/6J). Hepatocyte-specific *Kap1* KO mice were generated and genotyped according to [60] (strain C57BL/6J). Murine hepatoma cell line Hepa 1.6 cells were cultured using standard methods.

### Plasmids and lentiviral vectors

For KAP1 knockdown experiments, pLKO vector encoding shKAP1 and the empty vector as control were used. For KAP1 overexpression, pSicoR-KAP1-HA vector was used. 48 h after transduction, infected cells were selected with 1 μg mL^−1^ puromycin in growth medium for an additional 72 h. For de novo Kap1 excision, a non-integrating pHAGE2 Cre-IRES-PuroR lentiviral vector was used. Lentiviral vectors production protocols are available at http://tronolab.epfl.ch and backbones at Addgene (http://www.addgene.org).

### RT-PCR and RNA-Seq

Total RNA was extracted and DNase-I treated using a spin column-based RNA purification kit (Macherey-Nagel). cDNA was prepared with SuperScript II reverse transcriptase (Invitrogen). Primers were used for SYBR green qPCR (Applied Biosystems) and the sequences are provided in S2 File. For sequencing of mRNA (poly(A)+), 100-bp single-end RNA-seq libraries were prepared using the Illumina TruSeq Stranded or Unstranded mRNA reagents (Illumina). Cluster generation was performed with the resulting libraries using the Illumina TruSeq SR Cluster Kit v4 reagents. Sequencing was performed in 100-bp reads runs by Illumina HiSeq 2500. Further information about the mapping and analysis procedures is provided in S2 File.

### ChIP-qPCR and ChIP-Seq

ChIP and library preparation were done according to (Ecco, Cassano et al. 2016), with modifications as described in S2 File. Sequencing was performed in 100-bp reads run on Illumina HiSeq 2500. Primers sequences used for ChIP-qPCR are provided in S2 File.

### Bioinformatics analyses and statistics

R version 3.1.2 (http://www.R-project.org) or GraphPad Prism version 6.0 and 7.0 (http://www.graphpad.com) were used for statistical analyses and graphical representations of the data. Detailed bioinformatics analyses are provided in S2 File.

### Ethics statement

Experimental protocols were performed according to European Council Guidelines and the Swiss Federal Veterinary Office. Acceptable standards of human animal care and the experimental design of this study were approved by the Ethics Committee for Animal Care of the Vaud Region in Switzerland (licenses 25350 and 22919).

### Data access

All next-generation sequencing data have been submitted to the NCBI Gene Expression Omnibus (GEO) (http://www.ncbi.nlm.nih.gov/geo/) database under the accession number GEO: GSE87734.

## Supporting information

S1 Fig **(A)** Positional correlation between the 3’ end of MMSAT4-related transcripts and MMSAT4 elements, whether overlapping annotated genes or not. **(B)** (Top) Sequence alignment of *KZFP/rGU*s coming from three circumscribed stretches of DNA, located on chromosome 13 (cluster 5, 6_1 and 6_2 on the plot). The clusters are color-coded as follows: cluster 5 in yellow, cluster 6_1 in purple and cluster 6_2 in green. (Center) Separate alignments of *KZFP/rGU*s per region are depicted. (Bottom) Alignment of all *KZFP/rGU*s elements, keeping the initial color code. The sequence alignment, summarized by vertical bars on the left side of the plot, reconstitutes almost perfectly the initial boundaries between regions. **(C)** Sequence alignment of the *KZFP-rGU*s in clusters (top) 7, (middle) 14 and (bottom) 16. Color-coding of *KZFP-rGU*s reflects their imputed age, indicated on the vertical left axis. The conservation score is represented on top of each alignment. **(D)** Number of TEs located within *KZFP/rGU*s clusters compared to the expected one, estimated by counting the TEs falling within comparable borders after shuffling the clusters within the same chromosome 100 times. The same was performed for *OLFR* and *VMNR* clusters. P-values were obtained by Fisher’s exact test. **(E)** TE-enrichment analysis by subfamily in *KZFP/rGU*s clusters. For each cluster, the x-axis represents the proportion in the genome, while the proportion in the region is plotted on the y-axis. Larger dots depict subfamilies significantly over-represented in the region (p-value < 0.01). **(F)** Same as S1E Fig, for *OLFR* and *VMNR* clusters.
(EPS)

S2 Fig **(A)** Divergence analysis of recent duplication events within *KZFP/rGU*s clusters: three nearly identical genomic stretches of approximately 40 kb were delineated within cluster 2 (core segments 1, 2 and 3, delineated by blue boxes, > 98% using UCSC BLAT alignment tool). The homology score for nucleotides flanking the core segments dramatically dropped (sequences enclosed by purple and red boxes), while a conserved pattern of EREs spanning the core segments and their neighboring regions could still be identified, notably two ERVs (*IAPEz* and *RLTR19*) found upstream and a LINE (*L1Md F2*) immediately downstream. Sequences between *RLTR19* and *L1Md F2* were highly homologous, each containing two units composed of a KRAB domain and a *KZFP/rGU* in close proximity and same orientation. Conservation was higher between core segments 1 and 2, including downstream of *L1Md F2*, suggesting that they emerged through a more recent duplication event than the one responsible for core segment 3.
(EPS)

S3 Fig **(A)** KAP1 peaks enrichment in *KZFP/rGU*s compared to *OLFR* and *VMNR* genes clusters in ES cells, MEF and liver cells. Peak counts were normalized by the total number of nucleotides contained in the clusters. **(B)** Enrichment analysis of KAP1 peaks per *KZFP/rGU*s cluster in ES cells, MEFs and Hepa 1.6 cells. The actual number of peaks within each cluster is compared to the expected one, estimated by counting the peaks falling within the cluster borders after shuffling the peaks within the same chromosome 10’000 times. **(C)** UCSC Genome Browser view of *KZFP/rGU*s clusters 3 and 4. From the top, tracks for genes annotated in RefSeq and Ensembl, *KZFP/rGU*s, *KZFP/rGU*s clusters and KAP1 peaks in ES cells, MEFs and Hepa 1.6 cells are displayed. Both *KZFP/rGU*s clusters 3 and 4 correlate an increased KAP1 binding sites density, although changes in terms of targeted loci and density of targets are visible across tissues. **(D)** Evaluation of expression differences upon *Kap1* removal between *KZFP/rGU*s in clusters and the isolated counterparts for each tissue. **(E)** Sequence alignment of (left) *KZFP/rGU*s and (right) promoters of *KZP/rGU*s-containing transcripts of cluster 10. The conservation score is represented on top. **(F)** Normalized RNA-Seq coverage over *KZFP/rGU*s in ES cells, considering a flanking region of 1.5 kb upstream and 3.5 kb downstream of each element. Signal from WT cells is depicted in green, while that of *Kap1* KO cells in grey. All elements expressed are plotted, separating sense and antisense transcription, and *KZFP/rGU*s in and out of APC genes (left, top and bottom, respectively). (Right) UCSC Genome Browser view of strand-specific RNA-Seq signals in ES *Kap1* KO and WT cells (grey and green tracks, respectively). Below the RNA-Seq profiles, the following tracks were added: RefSeq genes, our de-novo transcripts annotation, *KZFP/rGU*s, KRAB-encoding sequences, KAP1 peaks in the same tissue and repeats as reported in RepeatMasker. Green vertical bars highlight two ERVs (RLTR4 and IAPEZ) and a *KZFP/rGU* targeted by KAP1 and upregulated upon *Kap1* removal. Despite their proximity, deregulation of these ERVs and the *KZFP/rGU* resulted from individual, oriented transcription. **(G)** Fraction of TEs upregulated upon *Kap1* KO in *KZFP/rGU*s compared to *OLFR* and *VMNR* genes clusters in ES cells, MEF and liver cells. **(H)** TEs targeted by KAP1 within *KZFP-rGU*s clusters in mES cells, MEFs and Hepa 1.6 cells. For each cluster, numbers of TE-targeting KAP1 peaks were normalized for total numbers of TEs from corresponding subclass. **(I)** Mean log2 of the expression fold change upon *Kap1* removal in MEF (left) and liver (right) cells of elements present in *KZFP/rGU*s clusters: *KZFP/rGU*s, ERVKs, ERV1s and the rest of TEs (“others”) (dots), as well as their total average (dashed line). The analysis was performed per cluster, with the number of elements in each category reported on the right (categories with fewer than 5 elements were not considered).
(EPS)

S4 Fig **(A)** Positional correlation between *KZFP/rGU*s and KAP1 and SETDB1 peaks, determined by ChIP-Seq enrichments, in Hepa 1.6 cells. A window of 5 kb on each side of the *KZFP/rGU*s was considered. **(B)** ChIP-PCR analysis of SETDB1 binding over *KZFP/rGU*s in WT and KAP1 KD Hepa 1.6 cells. KAP1 KD cells stably express a short hairpin targeting *Kap1* transcripts (shKAP1), while the WT counterpart were similarly selected for expressing the corresponding control vector (shEmpty). **(C)** Normalized H3K9me3 ChIP-Seq enrichment in MEF WT and *Kap1* KO cells over *KZFP/rGU*s upregulated or unaffected upon *Kap1* inactivation.
(EPS)

S5 Fig **(A)** Nucleotide fraction found beneath H3K27ac KO but not WT MEFs in *OLFR* or *VMNR* and *KZFP/rGU*s clusters. In each case we computed the same calculation excluding peaks matching APC gene promoters or newly annotated *KZFP/rGU*s promoters. The same analysis limited to clusters 3–6 and 10, being the most deregulated ones in *Kap1* KO cells, was performed. **(B)** Ratio of H3K27Ac-enriched *KZFP-rGU*s clusters-contained TEs in *Kap1* KO and in WT MEF cells, listed by subfamilies (with an inclusion threshold of at least 3 elements for a given subfamily).
(EPS)

S6 Fig **(A)** Expression of *KZFP/rGU*s in ES cells WT and *Kap1* KO based on their distance to a KAP1 binding site. We isolated *KZFP/rGU*s overlapping a KAP1 peak, those less than 5 kb, less than 20 kb and more than 20 kb away from a KAP1 targeted locus. Each element belongs exclusively to one category. **(B)** Normalized RNA-Seq coverage over *KZFP/rGU*s, considering a flanking region of 1.5 kb upstream and 3.5 kb downstream of each element. Elements dysregulated upon KAP1 depletion in MEFs are selected, and their coverage is plotted for WT cells, *Kap1* KO cells and *Kap1* KO cells complemented with a sh-resistant copy of *Kap1*. **(C)** *KZFP-rGU*s transcriptional changes detected by RT-qPCR upon de-novo KAP1 excision in MEF WT cells (top), and upon KAP1 complementation of *Kap1* KO cells (bottom), compared to their WT and stable *Kap1* KO counterparts.
(EPS)

S1 Table Table containing genomic coordinates of *KZFP/rGU*s clusters. (EPS)

S2 Table Table containing genomic coordinates of *OLFR* and *VMNR* genes clusters. (EPS)

S3 Table *KZFP/rGU*s database, listing: genomic sequences of *KZFP/rGU*s, orientation, cluster, coordinates of the cluster, coordinates of the relative transcript, orientation and ID of the transcript, whether it corresponds to an existing entry of the Ensembl version 67 annotated genes (with the Ensembl ID and the associated gene name), number of C2H2 ZNFs, whether a KRAB-encoding sequence is present in the same transcript, the ZNFs specificity residues and the amino acid sequence encoded by the transcript (until a STOP codon in the same reading frame). For detailed information on the analysis procedures followed to build this table, refer to S2 File.
(XLSX)

S4 Table Table containing normalized counts over *KZFP/rGU*s derived from RNA-Seq analyses performed in mESC, MEF and liver cells. (XLSX)

S1 File Gene transfer format (GTF) file of putative *KZFP/rGU*s-related ORFs. Their identifiers (“gene_id” and “transcript_id”) were attributed arbitrarily. For detailed information over the sequences, see S3 Table.
(GTF)

S2 File Supplemental Procedures. File containing detailed information about the experimental and data analysis procedures.
(DOCX)
