# Supplementary material for: The mouse genome displays highly dynamic populations of KRAB-zinc finger protein genes and related genetic units
Source: PLoS One. 2017 Mar 23;12(3):e0173746. doi: 10.1371/journal.pone.0173746 (PMC5363842; doi:10.1371/journal.pone.0173746)
Supplement: S2 File — File containing detailed information about the experimental and data analysis procedures. (DOCX) [file pone.0173746.s012.docx]

**Supplemental Procedures**

**Cell culture and cell-based assays.** *Kap1* KO and *loxP/loxP* control MEFs were generated and cultured as previously described [1][. For RNA-Seq experiments of MEFs, a stably-growing](#_ENREF_6) *Kap1* KO clone was selected and kept in culture for many passages, in parallel with its non-excised WT counterpart. De novo deletion of *Kap1* in these cells was done with transduction of a selectable Cre-expressing vector. For KAP1 ChIP experiments in MEFs, MEF *Kap1* KO cells were complemented with HA-tagged KAP1 with expression levels similar to the endogenous protein. ES cells were cultured as previously described [1, 2][, using the](#_ENREF_6) *Kap1 loxP/loxP* ES3 line or its *Kap1* conditional KO derivative after transduction with a tamoxifen-inducible Cre vector [3][. For KO induction, cells were treated overnight with 1μM tamoxifen (Sigma) and collected at the latest 4-5 days later.](#_ENREF_5) Hepatocyte-specific *Kap1* KO mice were generated and genotyped according to [4][.](#_ENREF_1) KAP1 KD was performed with selectable shRNA vectors targeting *Kap1* or the empty vector as control. Cells were selected with 1μg/mL puromycin when necessary.

**ChIP-PCR and ChIP-seq.** Cells were washed with PBS, fixed for 10 minutes at 1% formaldehyde, and quenched with glycine (at 125 mM final) for 5 minutes at room temperature. Cells were washed three times with ice-cold PBS, and harvested. The pellet was lysed, resuspended in 1 mL of sonication buffer (10 mM Tris at pH 8, 200 mM NaCl, 1 mM EDTA, 0.5 mM EGTA, 0.1% NaDOC, 0.5% NLS, and protease inhibitors), transferred to TC 12x12 tubes (Covaris), and sonicated (Covaris settings: 30 minutes, 5% duty cycle, 140W, 200 cycles). Chromatin was decrosslinked (RNAse A at 1μg/μL, 65°C overnight), purified and quantified by Nanodrop. Fragment size was assessed on a Bioanalyzer High Sensitivity chip (Agilent 2100). Immunoprecipitations were performed with 40 ug of chromatin (for KAP1 and SETDB1), or 20 ug of chromatin (for histone modifications), with antibody-coupled magnetic beads (Dynabeads, ThermoFisher) in IP buffer (10 mM Tris at pH 8.0, 100 mM NaCl, 1 mM EDTA, 0.5 mM EGTA, 2% Triton X-100, and protease inhibitors) overnight. Chromatin was decrosslinked (Proteinase K at 400ng/μL, 65°C overnight) and DNA purified for analysis. Antibodies used were HA (Covance, MMS- 101P), KAP1 (Trono laboratory, rabbit polyclonal S23470), H3K9me3 (Diagenode, C15410056), H3K4me1 (Diagenode, pAb-037-050), H3K27ac (Abcam, ab4729), SETDB1 [5][. ChIP samples were used for qPCR analysis by SYBER Green (Applied Biosystems) or for library preparation. Libraries of ChIP-ed DNA and the corresponding total input were performed with single-end adaptors according to](#_ENREF_7) (Rowe, Kapopoulou et al. 2013). [Sequencing runs were of 100 bp reads on Illumina HiSeq 2500 (Illumina).](#_ENREF_6) ChIP-Seq and ChIP-PCR experiments presented in this work are biological replicates.

**Primers sequences.**

| **Primer** | **Sequence** | **Purpose** |
| --- | --- | --- |
| Gapdh F | GCCCTTCTACAATGCCAAAG | ChIP-qPCR |
| Gapdh R | TTGTGATGGGTGTGAACCAC | ChIP-qPCR |
| Beta Tubulin F | GCACAGGATTGGGCTTTCA |  RT-qPCR |
| Beta Tubulin R | GAAATGTAGCCAATCAGACAAG | RT-qPCR |
| KAP1 F | CGGAAATGTGAGCGTGTTCTC | RT-qPCR |
| KAP1 R | CGGTAGCCAGCTGATGCAA | RT-qPCR |
| KZFP/rGU 1 F | GGA ATG TGG CAT CTC TTT GG | RT-qPCR/ChIP-qPCR |
| KZFP/rGU 1 R | CAA TGA GCA TGG GCA TAT CC | RT-qPCR/ChIP-qPCR |
| KZFP/rGU 2 F | AGA AGG TCG TGG AAC AAG | RT-qPCR/ChIP-qPCR |
| KZFP/rGU 2 R | TGG CAT CTC TGT GGT TAG | RT-qPCR/ChIP-qPCR |
| KZFP/rGU 3 F | ACT GAC AAA GGC AGT CTC | RT-qPCR/ChIP-qPCR |
| KZFP/rGU 3 R | TGG GAT GGT TGG GTA AAG | RT-qPCR/ChIP-qPCR |
| KZFP/rGU 4 F | CCA ACC ATC CCA TCT TAG | RT-qPCR/ChIP-qPCR |
| KZFP/rGU 4 R | GCA TTG GTA CGG TTT CTC | RT-qPCR/ChIP-qPCR |
| KZFP/rGU 5 F | GTG GGT TCC TTG ATT GAG AC | RT-qPCR/ChIP-qPCR |
| KZFP/rGU 5 R | CAC AGA GAT GCC ACT CTT C | RT-qPCR/ChIP-qPCR |
| KZFP/rGU promoter 1 F | GAA GCA CTG TTG AGC ACT ACT G | ChIP-qPCR |
| KZFP/rGU promoter 1 R | CTC CAC ATT GCC ACT GAT GAA G | ChIP-qPCR |
| KZFP/rGU promoter 2 F | GGA AGC ACA CTT CAA CCT TGA G | ChIP-qPCR |
| KZFP/rGU promoter 2 R | CTT TGT CTG ACT GGC ATG TCT G | ChIP-qPCR |
| KZFP/rGU promoter 3 F | TTC TGT CGC TAC TCT CAC | ChIP-qPCR |
| KZFP/rGU promoter 3 R | CCA AGG TTC TAC GAG GAT AC | ChIP-qPCR |
| KZFP/rGU promoter 4 F | GGT TGG TGT GAC TTG CTT TG | ChIP-qPCR |
| KZFP/rGU promoter 4 R | GGC AGT GTT CCC ATG CTT AC | ChIP-qPCR |

**Bioinformatics Analyses**

**Genomic and protein domains annotations.** MMSAT4 coordinates were extracted from RepeatMaster 3.2.8 and merged with a 2kb window size using a custom script. The merged file, listing 715 elements, was used throughout the study. MMSAT4 consensus sequence was obtained from Repbase (<http://www.girinst.org/repbase/>). BLAT and Table Browser tools of Genome Browser ([https://genome.ucsc.edu](https://genome.ucsc.edu/)) [6, 7] [were used for aligning sequences to the genome, retrieving the corresponding similarity percentages and manipulating bed files.](#_ENREF_7) Coordinates of protein-coding genes and pseudogenes were downloaded from Ensembl (<http://www.ensembl.org/index.html>), using the BioMart tool of the release number 67. C2H2 zinc finger protein and KRAB-encoding entries (code PF00096 en and PF01352 respectively) were downloaded from the same database. C2H2 or KRAB-domain encoding sequences were annotated in the genome according to Imbeault et al. [8][. Syntenic analysis of](#_ENREF_8) *KZFP* genes and pseudogenes was done as described in Imbeault et al. [8][.](#_ENREF_8)

**RNA-seq.** RNA-Seq reads were aligned to the mm9 genome assembly using HISAT 2 [9] [with default parameters (--rna-strandness R was added when it applied). Reads that were not uniquely mapped were discarded from the analysis using bamtools filter v2.4.1 with parameters -tag "NH:1". Gene counts were generated using the HTseq-count program with default parameters. TE counts were computed using the multicov script from the BEDtools software with the -split option. Only genes or TEs that had at least as many reads as samples present in the analysis were considered further. Sequencing depth normalization and differential expression analyses were performed using the voom function from the R package LIMMA](#_ENREF_9) [10] [from Bioconductor](#_ENREF_10) [11][. The gene library sizes as given by voom were used](#_ENREF_11) to normalize the TEs counts. P-values were computed using a moderated t-test and corrected for multiple testing using the Benjamini-Hochberg method [12][. To be considered significantly upregulated, a gene or a TE had to have 2-fold increased expression and an adjusted p-value lower than 0.05.](#_ENREF_12)

**ChIP-seq.** Reads were mapped using Bowtie 2 [13] [to the mm9 mouse genome assembly, with the sensitive-local mode. Peaks were called using MACS](#_ENREF_3) [14][, using total input as control and setting a cutoff score of 80.](#_ENREF_8)

**Bigwig files.** BigWigs files for the ChIP-seq experiments were generated using the Bedtools genomecov program followed by the bedGraphToBigWig program from the UCSC tools. The scaling factor option was set for each file in order to obtain a BigWig normalized to reads per 100 mio mapped reads.

**Coverage plots.** ChIP-seq or RNA-Seq signals over each feature of interest, to which the indicated flanking region was added, were extracted from the normalized bigwig files. For plotting, the lengths of the regions of interest were each scaled to a relative scale (0-100%).

**Heatmaps.** Heatmaps were generated using the heatmap.2 function of the R package gplots. Euclidean distances were used for the clustering and no scaling was applied on the rows, except for Fig. 3A where a Z-score was applied.

**Correlation studies.** Correlation analyses were performed with the online tool ChIP-Cor (<http://ccg.vital-it.ch/chipseq/chip_cor.php>) using input files in BED format. Histograms were displayed as raw counts or after global normalization (as specified in the plot’s y-axis).

**Circular genomic visualization.** The circlize package of R was used to plot the genomic map of MMSAT4 elements.

**Multiple alignments.** Multiple alignments of *KZFP/rGU*s were computed using MAFFT (v7.294b) with parameters –auto –ep 0.123. Phylogenic tree was computed using the web tool from *ClustalW2 Phylogeny* (<http://www.ebi.ac.uk/Tools/phylogeny/clustalw2_phylogeny/>). Multiple alignment of protein sequences were computed using Clustal Omega (<http://www.ebi.ac.uk/Tools/msa/clustalo/>). Plotting of the alignment was performed in python. For each alignment, a conservation score defined as the ratio of bases that are common throughout the sequences was computed and smoothed with a window of 75 bp before being plotted on top of the alignment.

**De-novo transcripts assembly**. De-novo transcriptome was assembled using stringtie (v.1.1.1) [15] [using parameters -c 1 and -f 0.01](#_ENREF_15) for a total of 6 mESC RNA-seq datasets (3 WT and 3 *Kap1* KO replicates). The resulting GTFs were merged per genotype using the merge functionality of the Stringtie program. The final transcriptome was derived from these files using the cuffmerge function of cufflinks v2.2.1. Bedtools was used to extract the *KZFP/rGU*s-containing transcripts (GTF file available in supplementary data Supplemental File S1) and the resulting subset was annotated, when possible, through the list of murine genes deposited on BioMart at Ensembl, version 67.

***KZFP/rGU*s protein sequence extraction and analysis.** Genomic sequences corresponding to *KZFP/rGU*s-related transcripts were extracted using the gffread function of cufflinks and translated in the three different reading frames using Biopython. For each *KZFP/rGUs*-related transcript, the reading frames yielding C2H2-containing aminoacid sequences not interrupted by STOP codons were selected and reported (none were reported in case of the absence of C2H2 sequence). We screened these proteins for the KRAB-encoding domain (Pfam entry PF01352) using hmmsearch function of the Homer v.3.1.b1 program with default parameters. For the annotations of C2H2-encoding motif, we used the regular expression “C.{2,5}C.{12}H.{3,5}H” in python. For each C2H2 ZF, the three DNA-contacting residues were extracted as the amino acids at positions -1, 3 and 6 of its alpha helix.

***KZFP/rGU*s database.** A table listing all the above-mentioned information relative to *KZFP-rGU*s-associated transcripts is available in the supplementary data (Supplementary Table S3).

**Statistical analysis.** Statistical difference was assessed by multiple t test (using Sidak-Bonferroni correction) for H3K4me1 ChIP-PCR enrichments and cluster fold changes, Fisher’s exact test for KAP1 peaks enrichment analysis and Mann-Whitney test for expression comparisons (represented as boxplots), with: p<0.0001: ****, p<0.001:***, p<0.01:**, p<0.05:*.

**Public sequencing data.** Raw reads were downloaded from publicly available ChIP-seq and RNA-seq ([GSE74278](http://www.ncbi.nlm.nih.gov/geo/query/acc.cgi?acc=GSE74278), GSE29413 at <https://www.ncbi.nlm.nih.gov/geo/> and SRX338012-SRR1028806 at http://sra.dnanexus.com) and analyzed as described above.

**Over-representation test.** All intersects were computed using BedTools intersect function with 1 bp overlap. For TE enrichment analysis, over-representation was established by hypergeometrical test. ‘Low complexity’ and ‘Simple Repeats’ were excluded from the analysis. For ChIP-seq peaks enrichment analysis, shuffling was done for 10’000 times within the same chromosome, and expected random values were determined by the function BedTool.randomstats(). For TEs enrichment in *KZFP/rGU*s and *OLFR/VMNR* clusters, the same method was applied with 100 random permutations of the clusters borders and significance was inferred using Fisher’s exact test as implemented in the BedTools fisher function.

**Supplemental References**

1. Rowe HM, Kapopoulou A, Corsinotti A, Fasching L, Macfarlan TS, Tarabay Y, Viville S, Jakobsson J, Pfaff SL, Trono D: TRIM28 repression of retrotransposon-based enhancers is necessary to preserve transcriptional dynamics in embryonic stem cells. *Genome Res* 2013, 23:452-461.

2. Rowe HM, Friedli M, Offner S, Verp S, Mesnard D, Marquis J, Aktas T, Trono D: De novo DNA methylation of endogenous retroviruses is shaped by KRAB-ZFPs/KAP1 and ESET. *Development* 2013, 140:519-529.

3. Rowe HM, Jakobsson J, Mesnard D, Rougemont J, Reynard S, Aktas T, Maillard PV, Layard-Liesching H, Verp S, Marquis J, et al: KAP1 controls endogenous retroviruses in embryonic stem cells. *Nature* 2010, 463:237-240.

4. Bojkowska K, Aloisio F, Cassano M, Kapopoulou A, Santoni de Sio F, Zangger N, Offner S, Cartoni C, Thomas C, Quenneville S, et al: Liver-specific ablation of Kruppel-associated box-associated protein 1 in mice leads to male-predominant hepatosteatosis and development of liver adenoma. *Hepatology* 2012, 56:1279-1290.

5. Sripathy SP, Stevens J, Schultz DC: The KAP1 corepressor functions to coordinate the assembly of de novo HP1-demarcated microenvironments of heterochromatin required for KRAB zinc finger protein-mediated transcriptional repression. *Mol Cell Biol* 2006, 26:8623-8638.

6. Kent WJ: BLAT--the BLAST-like alignment tool. *Genome Res* 2002, 12:656-664.

7. Karolchik D, Hinrichs AS, Furey TS, Roskin KM, Sugnet CW, Haussler D, Kent WJ: The UCSC Table Browser data retrieval tool. *Nucleic Acids Res* 2004, 32:D493-496.

8. Imbeault M, Helleboid P-Y, Trono D: KRAB-ZFPs contribute to the evolution of gene regulatory networks. *Nature* 2017, DOI 10.1038/nature21683

9. Kim D, Langmead B, Salzberg SL: HISAT: a fast spliced aligner with low memory requirements. *Nat Methods* 2015, 12:357-360.

10. Law CW, Chen Y, Shi W, Smyth GK: voom: Precision weights unlock linear model analysis tools for RNA-seq read counts. *Genome Biol* 2014, 15:R29.

11. Gentleman RC, Carey VJ, Bates DM, Bolstad B, Dettling M, Dudoit S, Ellis B, Gautier L, Ge Y, Gentry J, et al: Bioconductor: open software development for computational biology and bioinformatics. *Genome Biol* 2004, 5:R80.

12. Benjamini Y, and Hochberg, Y.: Controlling the False Discovery Rate: A Practical and Powerful Approach to Multiple Testing. *Journal of the Royal Statistical Society Series B (Methodological)* 1995, 57:289-300.

13. Langmead B, Salzberg SL: Fast gapped-read alignment with Bowtie 2. *Nat Methods* 2012, 9:357-359.

14. Zhang Y, Liu T, Meyer CA, Eeckhoute J, Johnson DS, Bernstein BE, Nusbaum C, Myers RM, Brown M, Li W, Liu XS: Model-based analysis of ChIP-Seq (MACS). *Genome Biol* 2008, 9:R137.

15. Pertea M, Pertea GM, Antonescu CM, Chang TC, Mendell JT, Salzberg SL: StringTie enables improved reconstruction of a transcriptome from RNA-seq reads. *Nat Biotechnol* 2015, 33:290-295.
